# Necrosis-Induced Sterile Inflammation Mediated by Interleukin-1α in Retinal Pigment Epithelial Cells

**DOI:** 10.1371/journal.pone.0144460

**Published:** 2015-12-07

**Authors:** Yang Liu, Kazuhiro Kimura, Tomoko Orita, Koh-Hei Sonoda

**Affiliations:** 1 Department of Ophthalmology, Yamaguchi University Graduate School of Medicine, Ube City, Yamaguchi, Japan; 2 Department of Ophthalmology, First Hospital of Jilin University, Jilin, PR China; Eye Hospital, Charité, GERMANY

## Abstract

Endogenous danger signals released from necrotic cells contribute to retinal inflammation. We have now investigated the effects of necrotic cell extracts prepared from ARPE-19 human retinal pigment epithelial cells (ANCE) on the release of proinflammatory cytokines and chemokines by healthy ARPE-19 cells. ANCE were prepared by subjection of ARPE-19 cells to freeze-thaw cycles. The release of various cytokines and chemokines from ARPE-19 cells was measured with a multiplex assay system or enzyme-linked immunosorbent assays. The expression of interleukin (IL)–1α and the phosphorylation and degradation of the endogenous nuclear factor–κB (NF-κB) inhibitor IκB-α were examined by immunoblot analysis. Among the various cytokines and chemokines examined, we found that ANCE markedly stimulated the release of the proinflammatory cytokine IL-6 and the chemokines IL-8 and monocyte chemoattractant protein (MCP)–1 by ARPE-19 cells. ANCE-induced IL-6, IL-8, and MCP-1 release was inhibited by IL-1 receptor antagonist and by an IKK2 inhibitor (a blocker of NF-κB signaling) in a concentration-dependent manner, but was not affected by a pan-caspase inhibitor (Z-VAD-FMK). Recombinant IL-1α also induced the secretion of IL-6, IL-8, and MCP-1 from ARPE-19 cells, and IL-1α was detected in ANCE. Furthermore, ANCE induced the phosphorylation and degradation of IκB-α in ARPE-19 cells. Our findings thus suggest that IL-1α is an important danger signal that is released from necrotic retinal pigment epithelial cells and triggers proinflammatory cytokine and chemokine secretion from intact cells in a manner dependent on NF-κB signaling. IL-1α is therefore a potential therapeutic target for amelioration of sterile inflammation in the retina.

## Introduction

Inflammation is one of the first responses of the body to danger and serves to maintain or restore tissue integrity [1]. The danger signals that induce inflammation include not only pathogens (pathogen-associated molecular patterns [PAMPs]) but also host-derived endogenous molecules produced or released as a result of cell death or injury (damage-associated molecular patterns [DAMPs]) [2]. DAMPs released by necrotic cells alert the innate immune system to impending tissue damage and initiate responses that lead to the removal of cell debris from necrotic tissue. Sustained or excessive activation of the immune system can be deleterious, resulting in maladaptive and irreversible changes to tissue structure and function [3]. Cell death and inflammation in the absence of infection (sterile inflammation) are important biological processes and are thought to play a central role in several retinal diseases including age-related macular degeneration (AMD), diabetic retinopathy, and retinal detachment, all of which can lead to irreversible blindness [4,5,6].

The retinal pigment epithelium (RPE) is the outermost layer of the retina and has many important functions in homeostasis of the eye and maintenance of normal vision. RPE cells thus support the survival and normal functioning of photoreceptors by contributing to the outer blood-retinal barrier and thereby controlling the exchange of nutrients, waste products, ions, and gases between the overlying photoreceptors and underlying choroidal blood vessels [7]. As the first line of defense against danger, the RPE also plays a key role in immune defense of the retina. RPE cells are able to sense DAMPs and to evoke inflammatory responses via the production of inflammatory mediators [8]. The induction of inflammatory responses by damaged RPE cells has been suggested to serve as an initial event in drusen biogenesis, a hallmark of the early phase of AMD [9]. RPE cell necrosis mediated by receptor-interacting protein kinase contributes to cell loss and DAMP-mediated inflammation in double-stranded RNA–induced retinal degeneration [6].

Members of the interleukin (IL)–1 family of cytokines play important roles in the regulation of immune and inflammatory responses to infection or sterile insults. IL-1α is a key danger signal released by necrotic cells that exerts effects on both innate and adaptive immunity [10]. Several DAMPs released from necrotic RPE cells have been identified, including high mobility group box 1 protein (HMGB1) and heat shock protein 90 [6,9]. However, the possible role of IL-1α in retinal inflammation associated with necrosis has remained unclear. We have now examined the effects of necrotic cell extracts prepared from the human RPE cell line ARPE-19 (ANCE) on the release of proinflammatory cytokines and chemokines by intact ARPE-19 cells. The possible role of IL-1α in such effects was also investigated.

## Materials and Methods

### Materials

Dulbecco’s modified Eagle’s medium–nutrient mixture F12 (DMEM-F12), penicillin, streptomycin, gentamicin, fetal bovine serum, and trypsin-EDTA were obtained from Invitrogen-Gibco (Rockville, MD), and 24-well culture plates, four-well chamber slides, and cell culture flasks were from Corning (Corning, NY). A Bio-Plex protein array system and Bio-Plex human cytokine assay were obtained from Bio-Rad (Hercules, CA), and recombinant human IL-1α, IL-1 receptor antagonist (IL-1ra), antibodies to IL-1α, as well as enzyme-linked immunosorbent assay (ELISA) kits for IL-6, IL-8, and monocyte chemoattractant protein (MCP)–1 were from R&D Systems (Minneapolis, MN). Horseradish peroxidase–conjugated donkey antibodies to rabbit immunoglobulin G (IgG) as well as antibodies to IκB-α and to phospho–IκB-α were obtained from Cell Signaling (Beverly, MA). An IκB kinase 2 (IKK2) inhibitor was obtained from Calbiochem (La Jolla, CA). Recombinant bovine insulin, cholera toxin, recombinant human epidermal growth factor, a protease inhibitor cocktail, a pan-caspase inhibitor (Z-VAD-FMK), as well as antibodies to β-actin and to cytokeratin 18 were obtained from Sigma-Aldrich (St. Louis, MO). An enhanced chemiluminescence (ECL) kit and nitrocellulose membranes were obtained from Amersham Pharmacia Biotech (Uppsala, Sweden). Antibodies to the p65 subunit of nuclear factor–κB (NF-κB) as well as normal mouse IgG were obtained from Santa Cruz Biotechnology (Santa Cruz, CA), and rhodamine-phalloidin, Alexa Fluor 488–labeled and 544-labeled goat antibodies to mouse or rabbit IgG, and Syto 59 were from Molecular Probes (Eugene, OR). Vectashield mounting medium was obtained from Vector Laboratories (Burlingame, CA).

### Cell Culture

ARPE-19 cells were obtained from American Type Culture Collection (Manassas, VA) and were cultured in DMEM-F12 supplemented with 10% fetal bovine serum, penicillin (100 U/ml), and streptomycin (100 μg/ml) [11]. The cells were maintained as stock cultures in 75-cm^2^ flasks and were passaged every 5 to 7 days by exposure to trypsin-EDTA at confluence and dilution at a ratio of 1:3 or 1:4. They exhibited no evidence of melanin pigmentation, and cells between passages 20 and 25 were used for experiments. Simian virus 40–immortalized human corneal epithelial (HCE) cells were obtained from RIKEN Biosource Center (Tokyo, Japan) and were passaged in supplemented hormonal epithelial medium (SHEM), which consists of DMEM-F12 supplemented with 15% heat-inactivated fetal bovine serum, bovine insulin (5 μg/ml), cholera toxin (0.1 μg/ml), recombinant human epidermal growth factor (10 ng/ml), and gentamicin (40 μg/ml) [12]. HCE cells between passages 18 and 20 were used for experiments. All cells were maintained at 37°C under a humidified atmosphere of 5% CO_2_.

#### Preparation of Necrotic Cell Extracts

Necrotic cell extracts were prepared as described previously [13,14]. Confluent monolayers of ARPE-19 or HCE cells were washed once with phosphate-buffered saline (PBS) and exposed to trypsin-EDTA for 3 min, after which the detached cells were collected in DMEM-F12, washed twice with PBS, resuspended at a density of 1 × 10^6^ cells/ml in DMEM-F12, and subjected to three rounds of rapid freezing in liquid nitrogen and thawing in a 37°C water bath. The cell lysates were then centrifuged at 120 × *g* for 10 min at 4°C, and the resulting supernatants were collected as ANCE.

### Assay of Cytokine and Chemokine Release

Assays were performed as described previously [15]. ARPE-19 or HCE cells cultured in 24-well plates until they achieved confluence were incubated first for 1 day with DMEM-F12 alone and then for an additional 24 h with necrotic cell extracts. For evaluation of the effects of inhibitors on ANCE-induced cytokine or chemokine release, serum-deprived ARPE-19 cells were incubated first for 1 h in the absence or presence of IL-1ra, Z-VAD-FMK, or IKK2 inhibitor and then for 24 h in the additional presence of ANCE. The culture supernatants were collected, centrifuged at 120 × *g* for 5 minutes to remove debris, and frozen at –80°C until subsequent measurement of cytokine and chemokine concentrations with the use of a Bio-Plex human cytokine assay system. Standard proteins were dissolved in DMEM-F12 for generation of standard curves. The concentrations of cytokines and chemokines in culture supernatants were calculated with the use of Bio-Plex Manager software 4.0. The concentrations of IL-6, IL-8, and MCP-1 in culture supernatants were also determined with the use of ELISA kits.

### Immunoblot Analysis

For immunoblot analysis of IκB-α, serum-deprived cells cultured in 24-well plates were incubated with ANCE for the indicated times at 37°C and then lysed in 100 μl of a solution containing 1% Nonidet P-40, 50 mM Tris-HCl (pH 7.4), 100 mM NaCl, 10 mM MgCl_2_, 1 mM dithiothreitol, 1 mM phenylmethylsulfonyl fluoride, and 1% protease inhibitor cocktail. The cell lysates (10 μg of protein) were subjected to SDS-polyacrylamide gel electrophoresis on a 10% gel. For immunoblot analysis of IL-1α, portions (20 μl) of ANCE or of culture supernatants of cells incubated with or without ANCE were similarly separated by gel electrophoresis. Separated proteins were transferred electrophoretically to a nitrocellulose membrane, nonspecific sites of which were then blocked before incubation of the membrane with primary antibodies (each at a 1:1000 dilution). Immune complexes were detected with secondary antibodies and ECL reagents.

### Immunofluorescence Staining

For immunostaining of cytokeratin 18, ARPE-19 cells grown on four-well chamber slides were washed twice with PBS, fixed for 15 min at room temperature with PBS containing 4% paraformaldehyde, washed three times with PBS containing 1% bovine serum albumin (BSA), and then incubated for 1 h at room temperature with PBS-BSA containing mouse monoclonal antibodies to cytokeratin 18 or normal mouse IgG (control). The cells were washed three times with PBS-BSA, incubated for 30 min at room temperature with Alexa Fluor 488– or 544-conjugated goat antibodies to mouse IgG, washed three times with PBS-BSA, and then incubated for 30 min at room temperature with rhodamine-phalloidin to stain F-actin and with Syto 59 to stain nuclei. For immunostaining of NF-κB, ARPE-19 cells grown on four-well chamber slides were incubated at 37°C first for 24 h in serum-free DMEM-F12 and then for 30 min with ANCE. The cells were then washed twice with PBS, fixed with 4% paraformaldehyde in PBS, and washed an additional three times with PBS before permeabilization with 100% methanol for 6 min at –20°C. Nonspecific adsorption of antibodies was blocked by incubation for 30 min with PBS containing 3% BSA, and the cells were then incubated at room temperature first for 1 h with rabbit polyclonal antibodies to the p65 subunit of NF-κB or normal rabbit IgG (control) and then for 30 min with Alexa Fluor 488–conjugated goat antibodies to rabbit IgG and with Syto 59. All cells were finally washed with PBS, mounted in Vectashield mounting medium, and observed with a laser-scanning confocal microscope (Axiovert200M; Carl Zeiss, Tokyo, Japan).

### Statistical Analysis

Data are presented as mean values ± SD. All experiments were performed with triplicates and repeated at least three times. Statistical analysis was performed with the Tukey-Kramer test or Dunnett’s multiple comparison test. A *P* value of <0.05 was considered statistically significant.

## Results

### Effects of ANCE on Cytokine and Chemokine Release from ARPE-19 Cells

**Fig 1:**
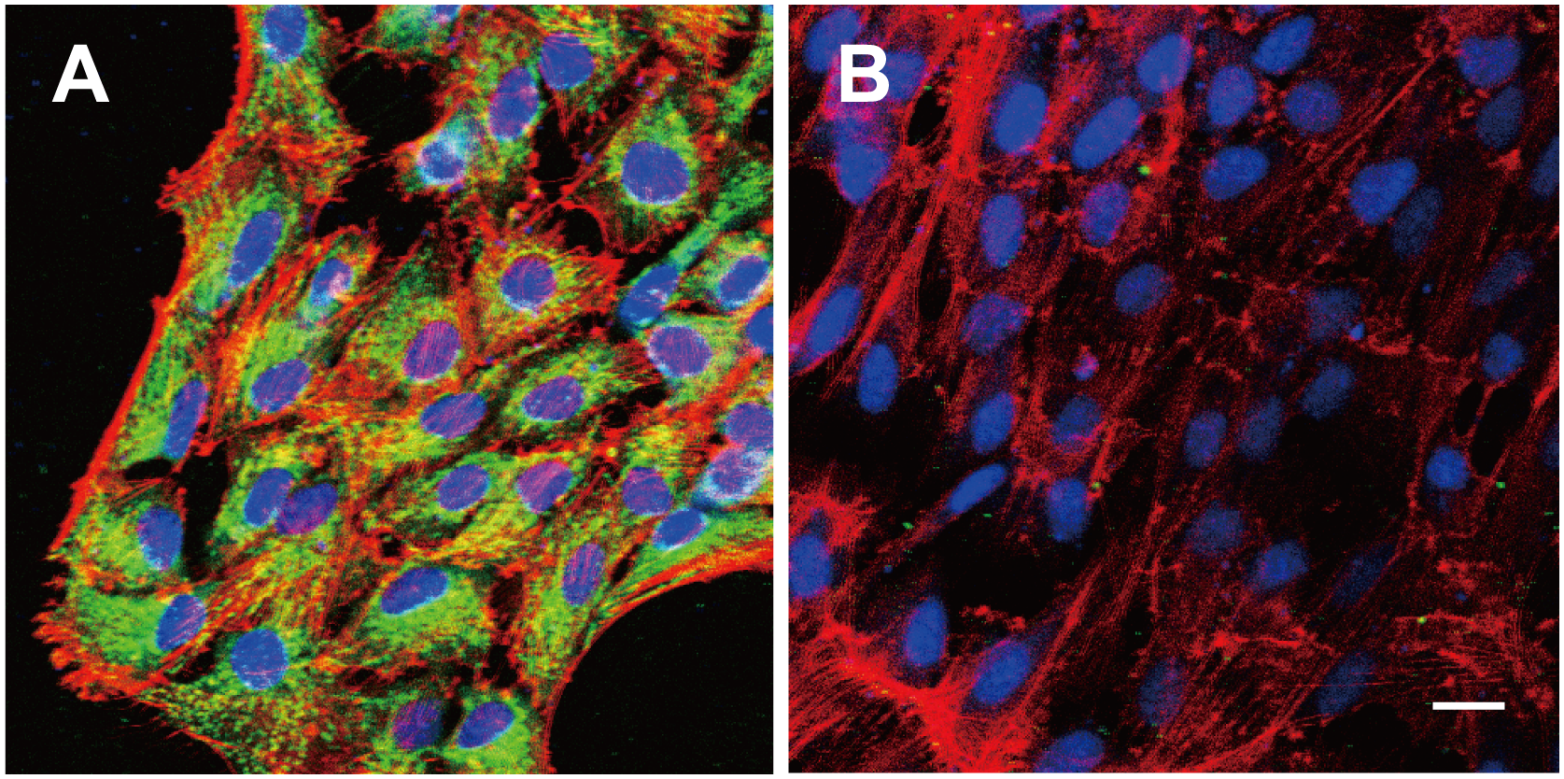
Expression of cytokeratin 18 by ARPE-19 cells. Cultured ARPE-19 cells were subjected to immunofluorescence staining (green) with antibodies to cytokeratin 18 (**A**) or with control mouse IgG (**B**). Cell nuclei and F-actin were stained with Syto 59 (blue) and rhodamine-phalloidin (red), respectively. Scale bar, 20 μm.

We first confirmed the purity of ARPE-19 cell cultures by examination of cell morphology and reactivity with antibodies to cytokeratin 18. Immunofluorescence staining revealed that all the cells were positive for cytokeratin 18 (Fig 1), and no changes in cell morphology or cytokeratin 18 immunoreactivity were apparent during culture for up to 20 to 25 passages.

Various inflammatory mediators released by RPE cells contribute to retinal inflammation [16]. To examine whether necrotic RPE cells are able to activate healthy RPE cells, we determined the effects of ANCE on the release of cytokines and chemokines from ARPE-19 cells with the use of a multiplex assay system. The cells were incubated in serum-free medium for 24 h before exposure to ANCE for 24 h, after which the culture supernatants were collected for analysis. The concentrations of IL-6, IL-8, and MCP-1 in culture supernatants of cells exposed to ANCE were markedly increased compared with those in culture supernatants of cells incubated without ANCE or with those in ANCE itself (Table 1). In contrast, ANCE had no substantial effect on the release of the other cytokines and chemokines examined (Table 1).

**Table 1:** Effects of ANCE on the release of cytokines and chemokines from ARPE-19 cells. Serum-deprived cells were incubated for 24 h in serum-free medium (SF) or with ANCE, after which culture supernatants were collected for measurement of cytokine and chemokine concentrations with a multiplex assay system. The concentrations of the analytes were also measured in ANCE alone.

Analyte	Cells + SF	Cells + ANCE	ANCE
IL-1β	0.01 ± 0.02	1.19 ± 0.16	1.51 ± 0.12
IL-1α	0.55 ± 0.28	10.99 ± 0.38	9.09 ± 0.62
IL-1ra	1.39 ± 0.68	34.36 ± 2.88	33.55 ± 2.27
IL-2	0.97 ± 0.77	13.51 ± 0.78	8.35 ± 2.54
IL-4	ND	3.67 ± 0.32	4.72 ± 0.43
IL-5	0.16 ± 0.06	0.52 ± 0.11	1.12 ± 0.07
**IL-6**	2.92 ± 0.94	**1014.29 ± 68.19** *	55.09 ± 7.45
IL-7	0.69 ± 0.25	12.50 ± 1.65	26.02 ± 2.46
**IL-8**	12.41 ± 10.9	**30,303.36 ± 2277.69** *	184.72 ± 26.11
IL-9	0.74 ± 0.39	8.13 ± 1.18	6.11 ± 0.61
IL-10	1.14 ± 0.36	11.61 ± 1.84	5.68 ± 0.83
IL-12 (p70)	7.65 ± 1.85	135.00 ± 13.42	36.65 ± 0.3
IL-13	0.43 ± 0.21	7.44 ± 0.68	4.46 ± 0.17
IL-15	0.41 ± 0.30	18.63 ± 1.32	17.35 ± 0.66
IL-17	0.62 ± 0.60	6.05 ± 1.61	4.67 ± 1.31
Eotaxin	6.45 ± 5.09	26.29 ± 3.39	17.94 ± 5.09
G-CSF	0.24 ± 0.24	17.3 ± 1.29	16.65 ± 1.04
GM-CSF	0.60 ± 0.25	18.42 ± 2.63	12.26 ± 4.36
IFN-γ	ND	66.85 ± 6.98	93.83 ± 3.89
IP-10	1.13 ± 1.30	23.87 ± 3.74	30.62 ± 3.38
**MCP-1**	39.28 ± 2.96	**3,444.83 ± 721.20** *	174.18 ± 3.82
MIP-1α	0.27 ± 0.31	1.95 ± 0.09	2.13 ± 0.22
MIP-1β	0.14 ± 0.18	12.97 ± 0.46	11.74 ±1.95
RANTES	1.7 7± 1.155	7.78 ± 0.33	7.92 ± 1.11
TNF-α	0.74 ± 0.62	17.00 ± 2.90	17.66 ± 2.15

Data are expressed as picograms per milliliter and are mean values ± SD from three independent experiments.

*P < 0.01 versus the corresponding values for cells cultured without ANCE or for ANCE alone (Dunnett’s test).

Abbreviations not defined in text: G-CSF, granulocyte colony-stimulating factor; GM-CSF, granulocyte-macrophage colony-stimulating factor; IFN-γ, interferon-γ; IP-10, IFN-γ–induced protein 10; MIP, macrophage inflammatory protein; RANTES, regulated on activation normal T expressed and secreted; TNF-α, tumor necrosis factor–α; ND, not detected.

### IL-1α in ANCE Is Required for ANCE-Induced IL-6, IL-8, and MCP-1 Release by ARPE-19 Cells

**Fig 2:**
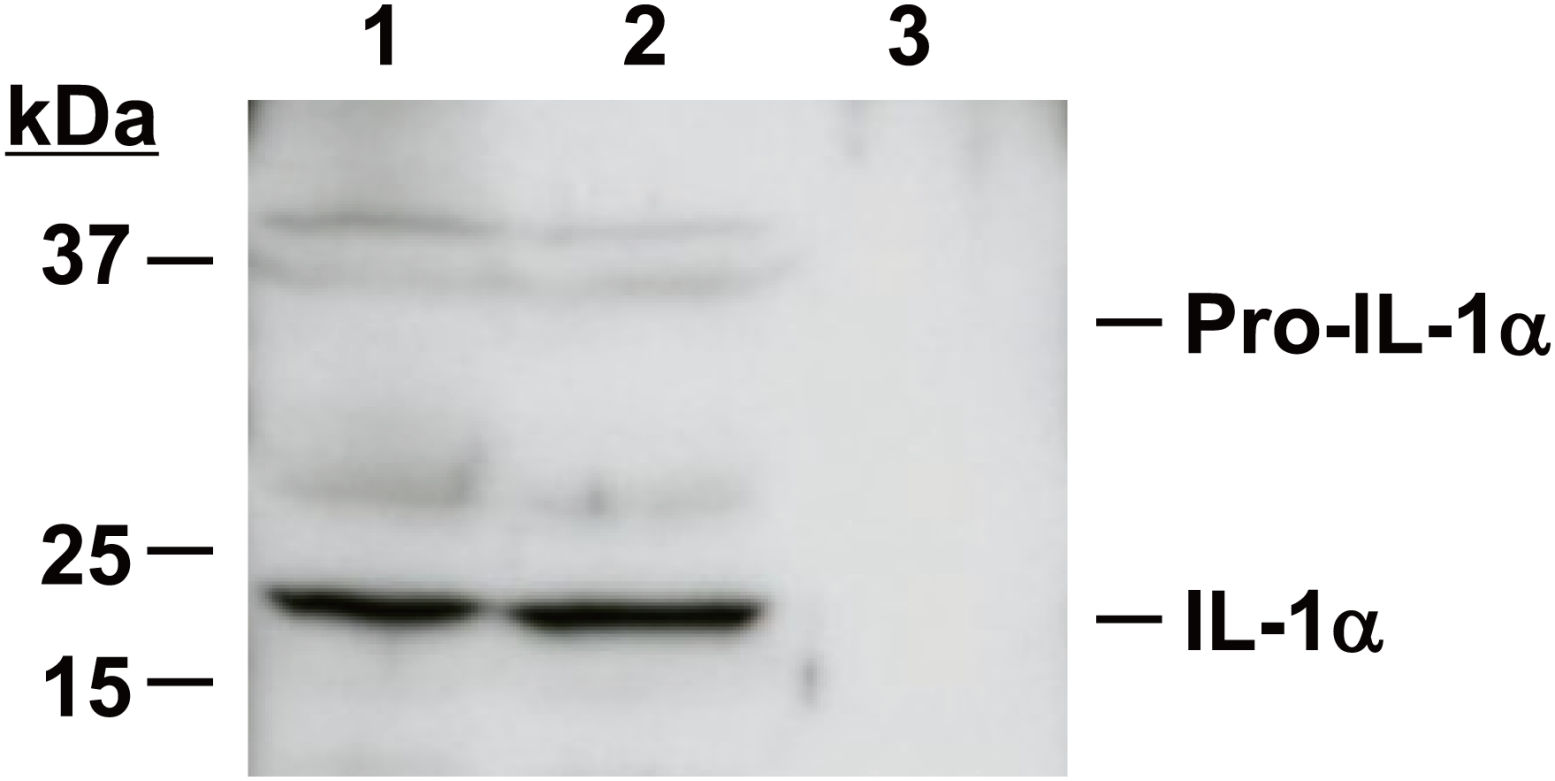
Immunoblot analysis of IL-1α in ANCE. Equal volumes of ANCE (lane 1) or of culture supernatants from serum-deprived ARPE-19 cells incubated for 24 h either with ANCE (lane 2) or in serum-free medium (lane 3) were subjected to immunoblot analysis with antibodies to IL-1α. Data are representative for three independent experiments.

IL-1α is a key danger signal released from necrotic cells [14], and we detected this cytokine in ANCE with the multiplex assay system (Table 1). To investigate whether IL-1α might contribute to ANCE-induced cytokine and chemokine release from ARPE-19 cells, we first examined ANCE for the presence of IL-1α protein by immunoblot analysis. Consistent with the results of the multiplex assay (Table 1), similar amounts of IL-1α were apparent in ANCE and in the culture supernatants of cells cultured with ANCE for 24 h (Fig 2). The pro form of IL-1α was detected at only low levels in these samples, however (Fig 2). Neither IL-1α nor pro–IL-1α was apparent in the culture supernatants of cells incubated with serum-free medium alone (Fig 2).

**Fig 3:**
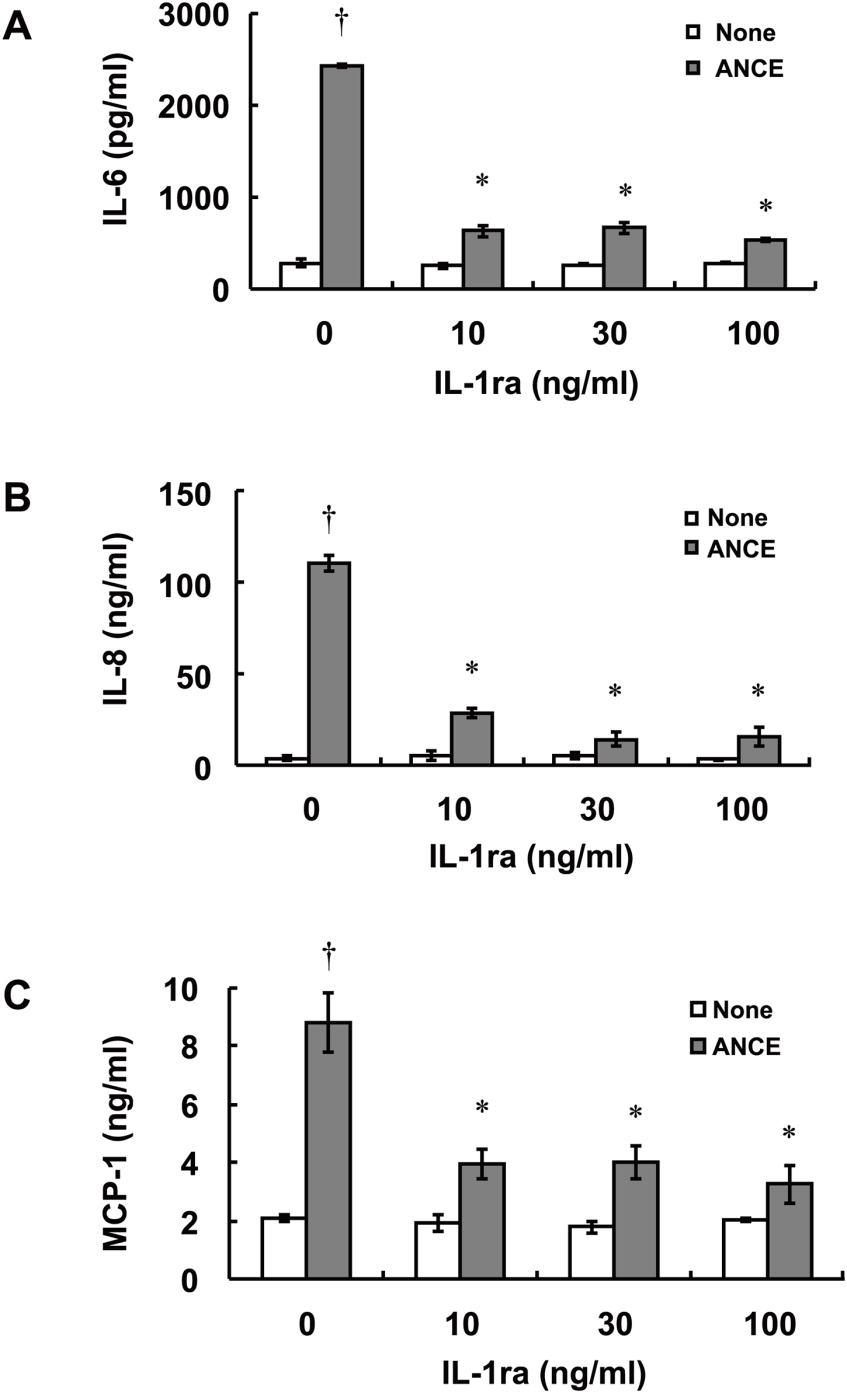
Effects of IL-1ra on ANCE-induced IL-6, IL-8, and MCP-1 release by ARPE-19 cells. Serum-deprived cells were incubated first for 1 h with various concentrations of IL-1ra and then for 24 h in the additional absence or presence of ANCE, after which the release of IL-6 (**A**), IL-8 (**B**), and MCP-1 (**C**) into the culture medium was measured. Data are mean values ± SD of triplicates from an experiment that was repeated a total of three times with similar results. †*P* < 0.01 versus the corresponding value for cells cultured without ANCE; *P < 0.01 versus the corresponding value for cells cultured with ANCE alone (Tukey-Kramer test).

IL-1ra inhibits the biological effects of IL-1 by binding to the IL-1 receptor [17]. We therefore next examined the effects of IL-1ra on ANCE-induced IL-6, IL-8, and MCP-1 release by ARPE-19 cells. The cells were exposed to IL-1ra (10 to 100 ng/ml) for 1 h before incubation for 24 h in the additional presence of ANCE. The ANCE-induced release of IL-6, IL-8, and MCP-1was inhibited by 74%, 74%, and 55%, respectively, by IL-1ra at a concentration of 10 ng/ml, by 72%, 87%, and 55%, respectively, at a concentration of 30 ng/ml, and by 78%, 87%, and 63%, respectively, at a concentration of 100 ng/ml (Fig 3).

**Fig 4:**
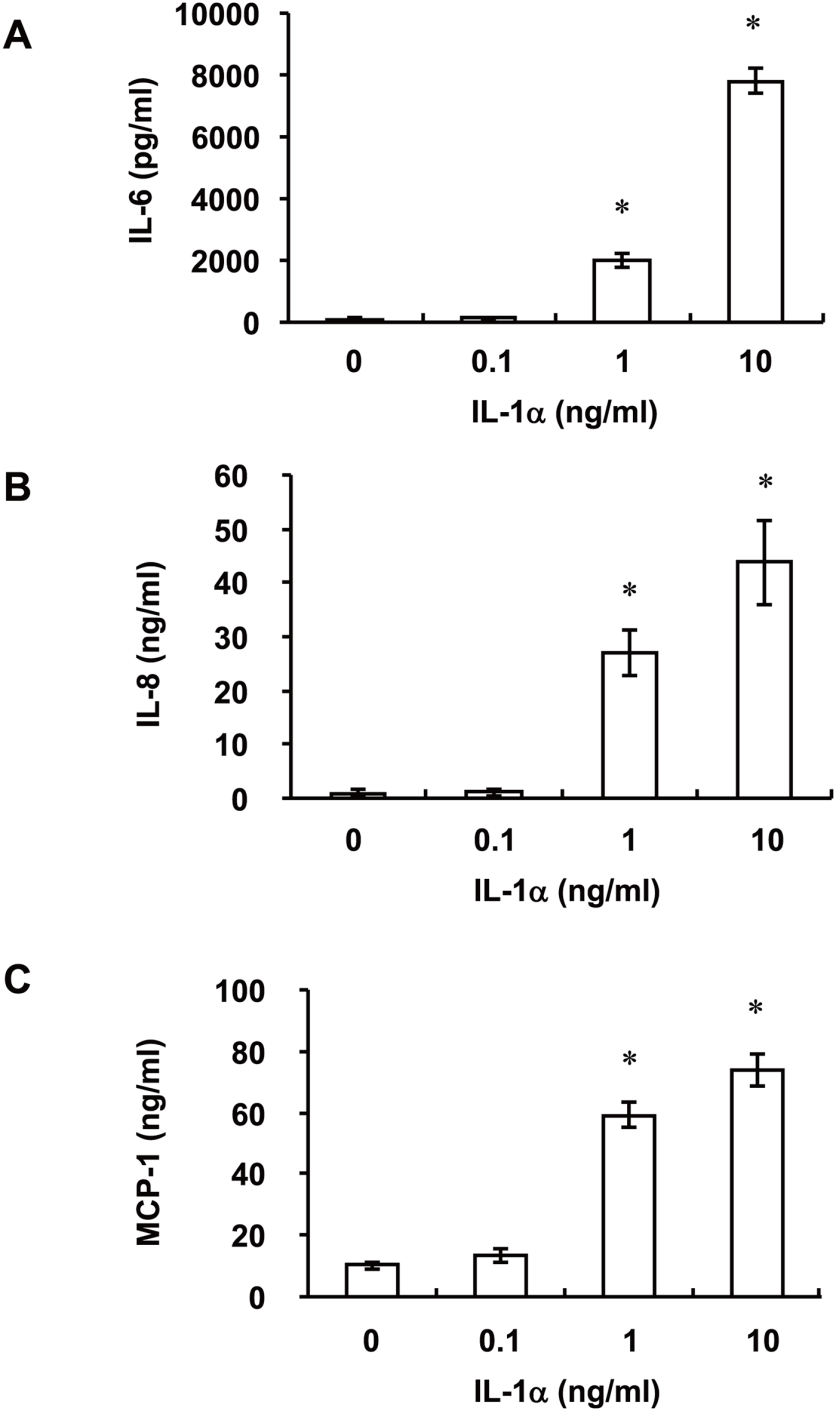
Effects of recombinant IL-1α on IL-6, IL-8, and MCP-1 release by ARPE-19 cells. Serum-deprived cells were incubated for 24 h with various concentrations of IL-1α, after which the release of IL-6 (**A**), IL-8 (**B**), and MCP-1 (**C**) into the culture medium was measured. Data are mean values ± SD of triplicates from an experiment that was repeated a total of three times with similar results. *P < 0.05 versus the corresponding value for cells cultured without IL-1α ((Dunnett’s test).

To confirm that IL-1α has the potential to function as a danger signal in sterile inflammatory responses of RPE cells, we examined the effects of recombinant human IL-1α on IL-6, IL-8, and MCP-1 production by ARPE-19 cells. Incubation of the cells for 24 h with IL-1α (0.1 to 10 ng/ml) induced the release of IL-6, IL-8, and MCP-1 in a concentration-dependent manner (Fig 4).

**Fig 5:**
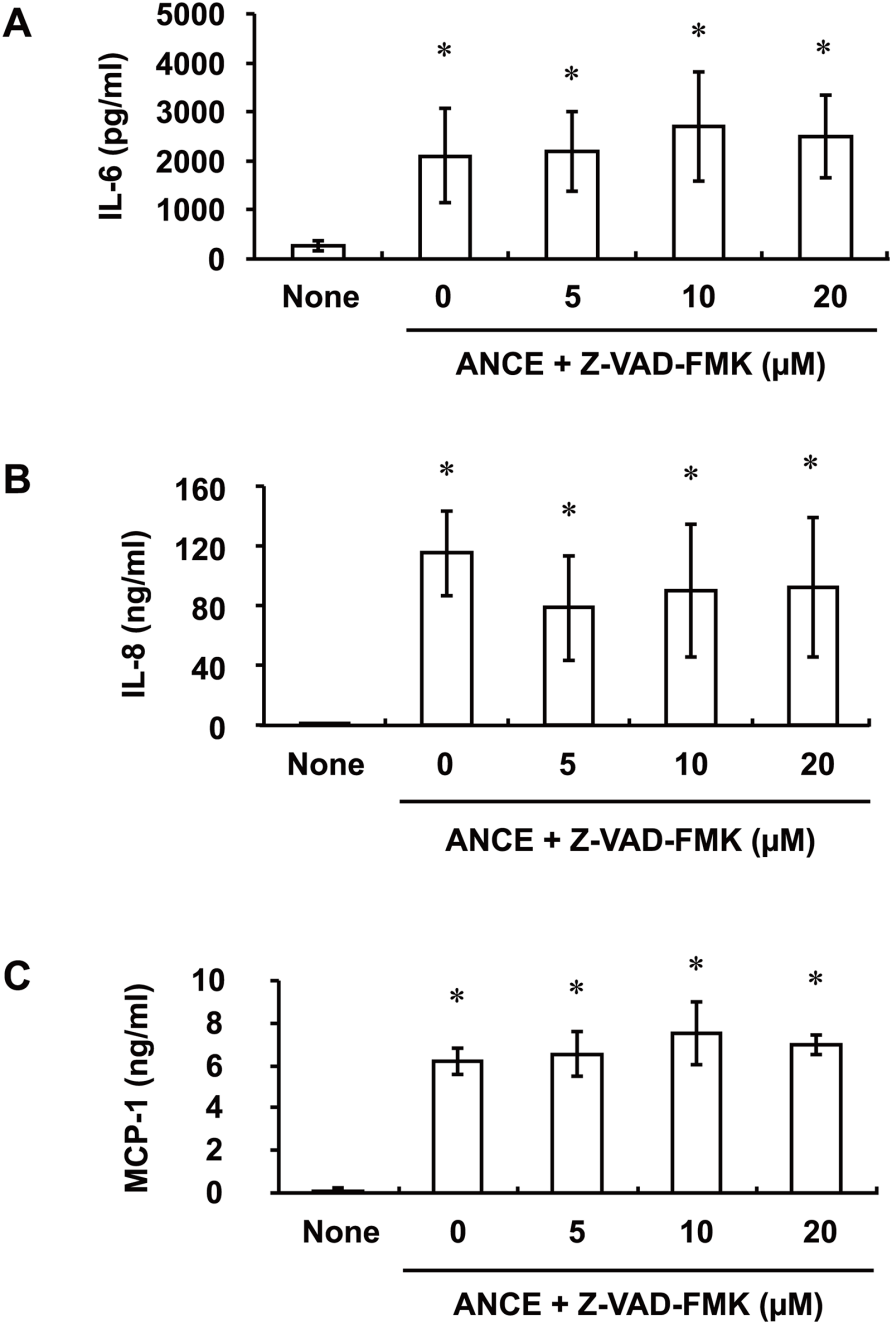
Effects of a pan-caspase inhibitor (Z-VAD-FMK) on ANCE-induced IL-6, IL-8, and MCP-1 release by ARPE-19 cells. Serum-deprived cells were incubated first for 1 h with various concentrations of Z-VAD-FMK and then for 24 h in the additional absence or presence of ANCE, after which the release of IL-6 (**A**), IL-8 (**B**), and MCP-1 (**C**) into the culture medium was measured. Data are mean values ± SD of triplicates from an experiment that was repeated a total of three times with similar results. *P < 0.05 versus the corresponding value for cells cultured without ANCE (Tukey-Kramer test).

Both IL-1α and IL-1β are endogenous mediators of the inflammatory response to cell injury. The pro form of IL-1β is cleaved by caspase-1 to generate the active cytokine [18]. Although it was present at only low levels in ANCE (Table 1), we investigated whether IL-1β contributes to ANCE-induced cytokine and chemokine expression in ARPE-19 cells by examining the effects of the pan-caspase inhibitor Z-VAD-FMK on ANCE action. Exposure of the cells to Z-VAD-FMK (5 to 20 μM) for 1 h before stimulation with ANCE for 24 h had no effect on the ANCE-induced release of IL-6, IL-8, and MCP-1 (Fig 5).

### Role of NF-κB Signaling in ANCE-Induced IL-6, IL-8, and MCP-1 Expression by ARPE-19 Cells

**Fig 6:**
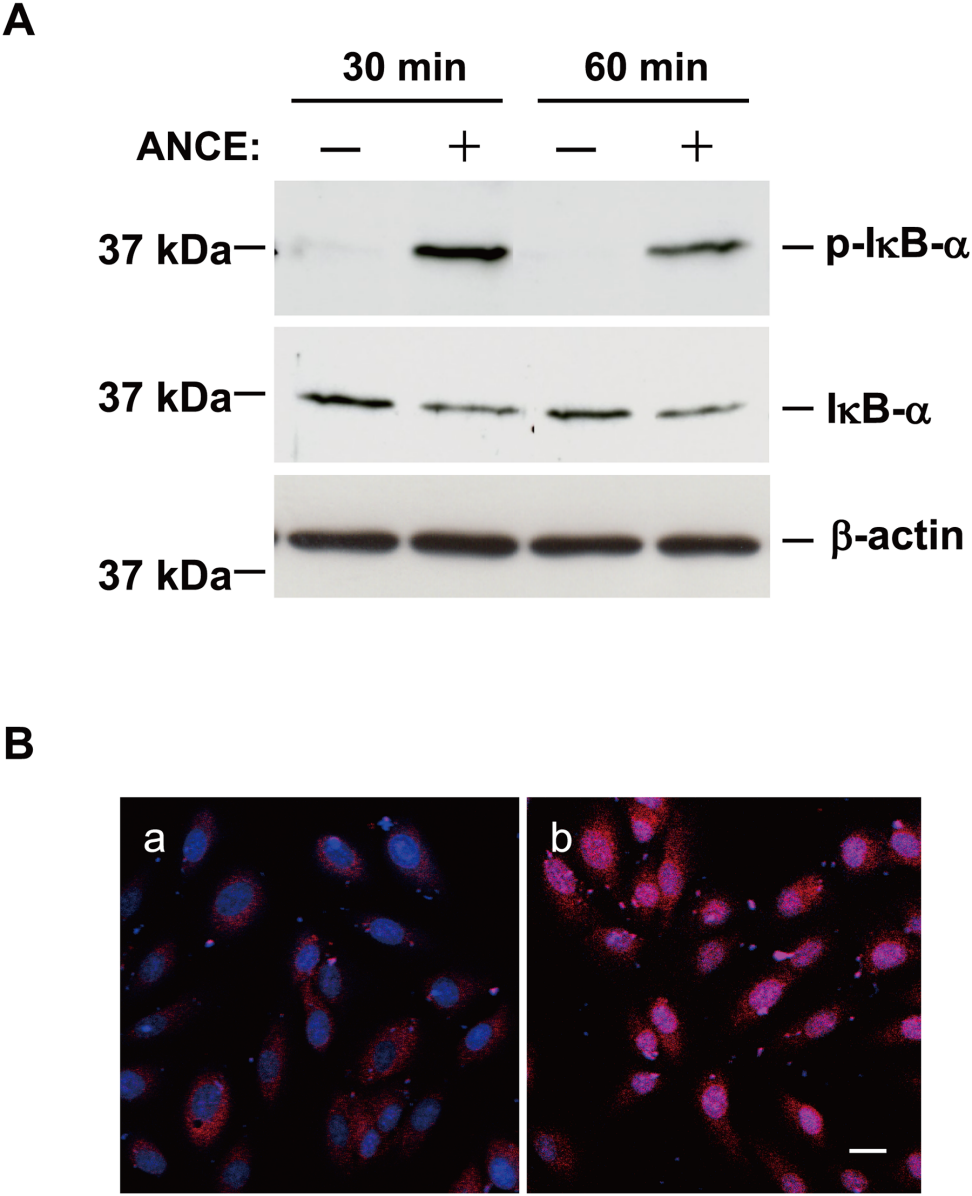
Effect of ANCE on the activation status of NF-κB in ARPE-19 cells. (**A**) Serum-deprived cells were incubated for 30 or 60 min in the absence or presence of ANCE, after which cell lysates were subjected to immunoblot analysis with antibodies to total or phosphorylated (p-) forms of IκB-α. The abundance of β-actin was examined as an internal control. (**B**) Serum-deprived cells were incubated for 30 min in the absence (panel a) or presence (panel b) of ANCE and were then fixed, permeabilized, and subjected to immunofluorescence analysis with antibodies to the p65 subunit of NF-κB (red). Nuclei were stained with Syto 59 (blue). Scale bar, 20 μm. All data are representative for three independent experiments.

**Fig 7:**
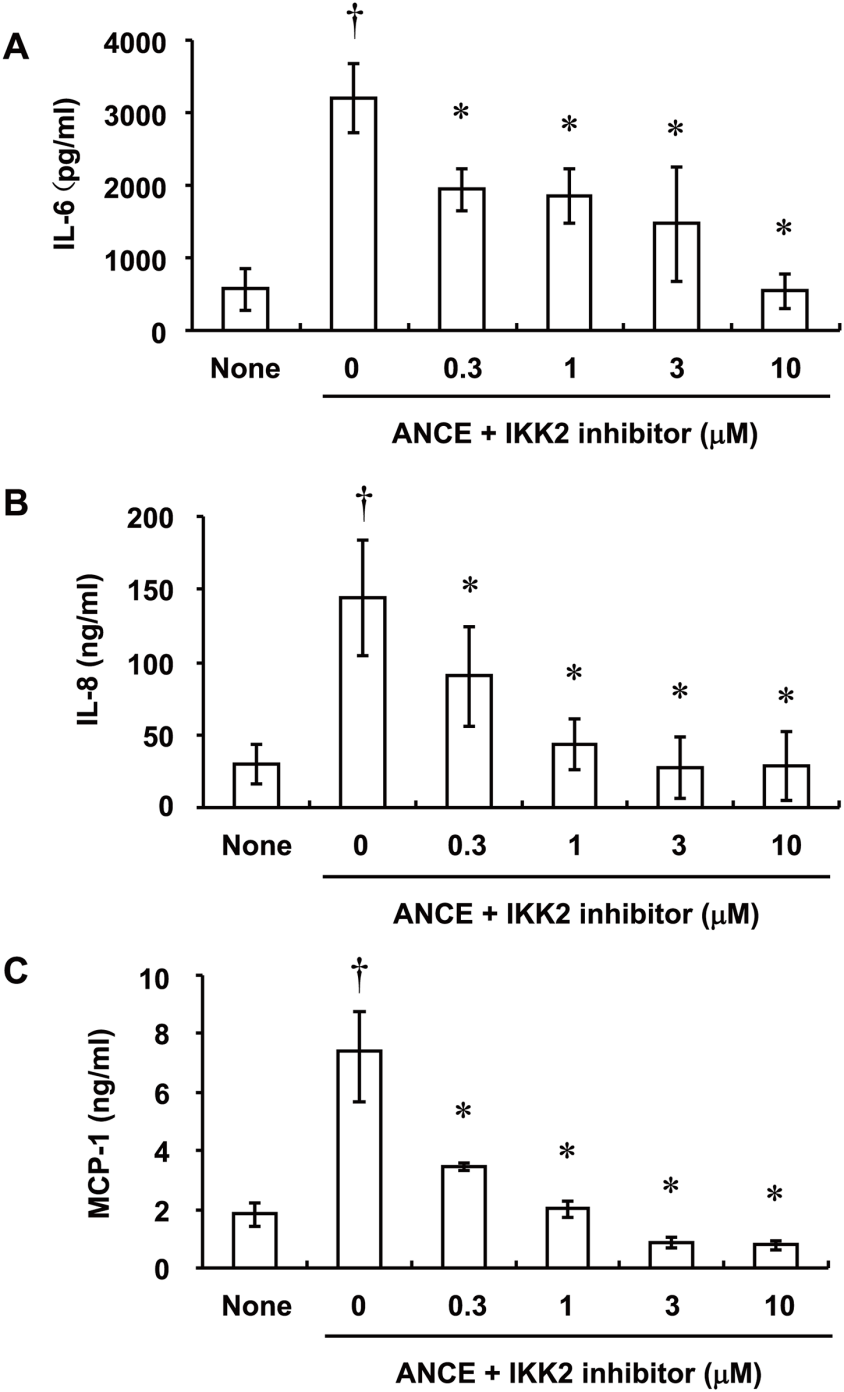
Effects of an IKK2 inhibitor on ANCE-induced IL-6, IL-8, and MCP-1 release from ARPE-19 cells. Serum-deprived cells were incubated first for 1 h with various concentrations of IKK2 inhibitor and then for 24 h in the additional absence or presence of ANCE, after which the release of IL-6 (**A**), IL-8 (**B**), and MCP-1 (**C**) into the culture medium was measured. Data are mean values ± SD of triplicates from an experiment that was repeated a total of three times with similar results. †*P* < 0.01 versus the corresponding value for cells cultured without ANCE; *P < 0.05 versus the corresponding value for cells cultured with ANCE alone (Tukey-Kramer test).

Given that signaling by the transcription factor NF-κB is implicated in the induction of genes for proinflammatory cytokines and chemokines in various cell types [19], we examined whether ANCE might affect the phosphorylation or abundance of the endogenous NF-κB inhibitor IκB-α in ARPE-19 cells. Immunoblot analysis revealed that ANCE induced both the phosphorylation and degradation of IκB-α, with both effects being apparent at 30 and 60 min after the onset of stimulation (Fig 6A). Immunofluorescence analysis also showed that incubation of ARPE-19 cells with ANCE for 30 min induced translocation of the p65 subunit of NF-κB from the cytosol to the nucleus (Fig 6B). In addition, exposure of the cells to an IKK2 inhibitor (which blocks NF-κB signaling) attenuated ANCE-induced IL-6, IL-8, and MCP-1 release in a concentration-dependent manner (Fig 7), with these effects being statistically significant at 0.3 μM and maximal at 10 μM.

### Effects of HNCE on Cytokine and Chemokine Release from HCE Cells

**Fig 8:**
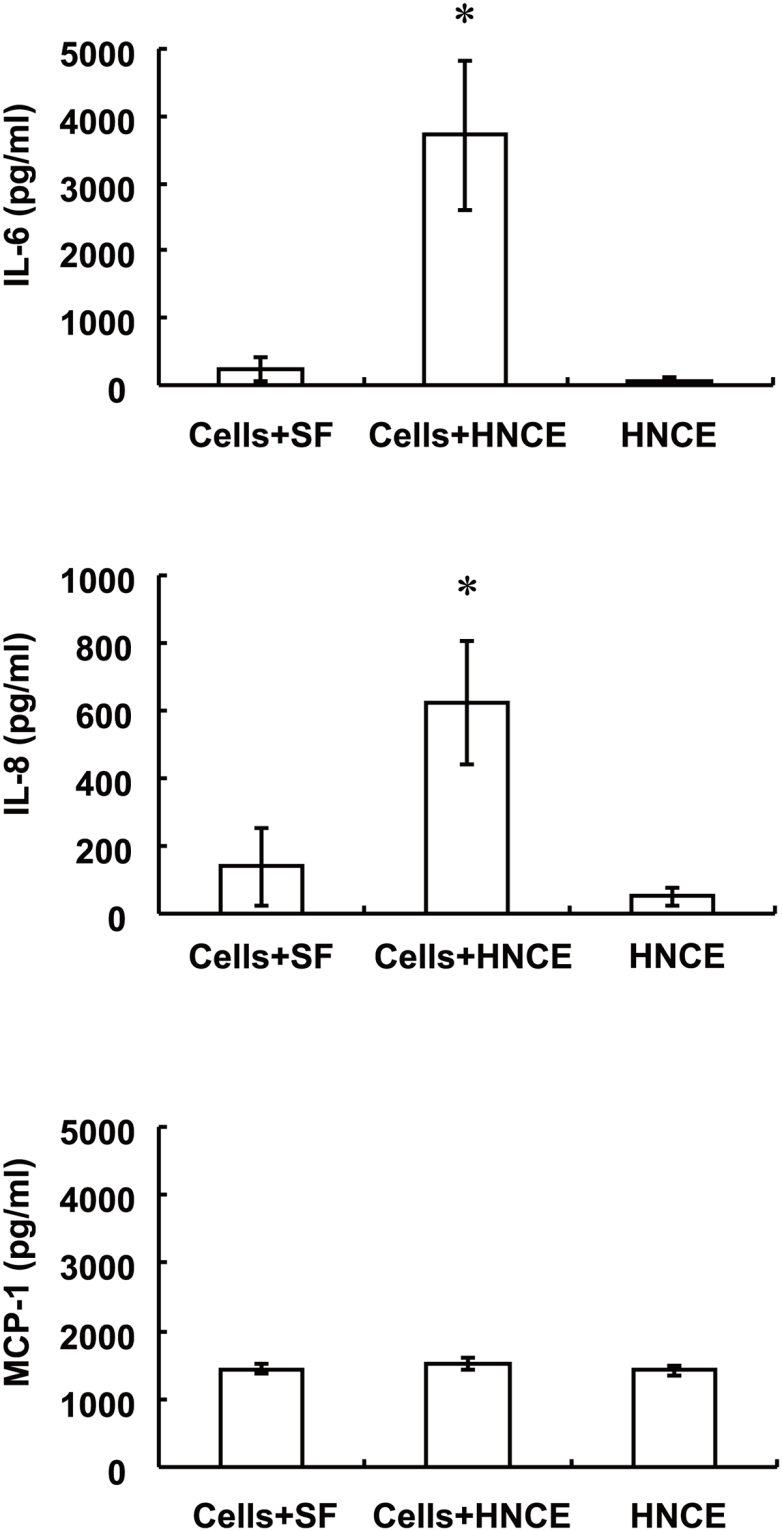
Effects of HNCE on IL-6, IL-8, and MCP-1 release by HCE cells. Serum-deprived HCE cells were incubated for 24 h in serum-free medium (SF) or with HNCE, after which the release of IL-6 (**A**), IL-8 (**B**), and MCP-1 (**C**) into the culture medium was measured. HNCE alone was similarly analyzed. Data are mean values ± SD of triplicates from an experiment that was repeated a total of three times with similar results. *P < 0.05 versus the corresponding value for cells incubated in serum-free medium or for ANCE alone (Dunnett’s test).

Finally, as a control, we determined the effects of necrotic cell extracts prepared from HCE cells (HNCE) on the release of IL-6, IL-8, and MCP-1 from HCE cells. The cells were incubated in serum-free medium for 24 h before exposure to HNCE for 24 h, after which the culture supernatants were collected for analysis. The concentrations of IL-6 and IL-8 in culture supernatants of cells exposed to HNCE were significantly increased compared with those in culture supernatants of cells incubated without HNCE or with those in HNCE itself (Fig 8). In contrast, HNCE had no effect on the release of MCP-1 (Fig 8).

## Discussion

We have here shown that, among the various cytokines and chemokines examined, ANCE markedly stimulated the release of IL-6, IL-8, and MCP-1 from ARPE-19 cells. These effects of ANCE were attenuated by IL-1ra and by an IKK2 inhibitor, but not by a pan-caspase inhibitor. Furthermore, ANCE induced the phosphorylation and degradation of IκB-α as well as triggered translocation of the p65 subunit of NF-κB from the cytosol to the nucleus in ARPE-19 cells.

Cytokines and chemokines play key roles in ocular immune and inflammatory responses through regulation of the activation, infiltration, and proliferation of immunocompetent cells. IL-6 is a pleiotropic cytokine that mediates the acute-phase response and contributes to the regulation of lymphocyte function [20]. IL-8 and MCP-1 are potent and selective chemoattractants for neutrophils and monocytes-macrophages, respectively. Elevated levels of IL-6, IL-8, and MCP-1 in aqueous humor have been found to be associated with diabetic macular edema and to serve as predictors of this disease [21]. IL-6 and IL-8 have also been associated with the risk for AMD [22]. Necrotic cells were previously shown to induce IL-6 secretion from intact mesothelial cells and thereby to trigger recruitment of neutrophils [13]. In addition, necrotic cells induced the release of IL-6 and MCP-1 from vascular smooth muscle cells [23]. Consistent with these findings, we have now shown that ANCE induced pronounced increases in the release of IL-6, IL-8, and MCP-1 by ARPE-19 cells, suggesting that these three inflammatory molecules are key mediators of sterile inflammation in the retina. RPE cells perform a variety of complex functions that are essential for proper visual function, and they also play a central role in immune defense in the retina. Dysfunction of the RPE has detrimental consequences and precedes photoreceptor atrophy in several eye diseases, including AMD [24]. Our finding that RPE cells released inflammatory cytokines and chemokines in response to necrotic cell extracts suggests that RPE cell damage may promote sterile inflammation in retinal diseases.

DAMPs are endogenous proteins that can induce inflammation and exacerbate tissue damage following their release during tissue injury [25]. Many DAMPs, including several heat shock proteins, S100 proteins, HMGB1, and hyaluronan-like proteins, have been detected in the human vitreous [26]. Breakdown of these proteins during inflammation can also further activate inflammatory pathways, as has been demonstrated in diabetes and mouse models of retinal degeneration [6,27]. IL-1 family members are important mediators of diverse types of inflammation. Both IL-1α and IL-1β bind to the same receptor to induce identical biological actions. IL-1ra inhibits the biological activities of IL-1 by also binding to the IL-1 receptor [17]. Both IL-1α and IL-1β are synthesized by RPE cells (Table 1) [28,29]. In the present study, IL-1ra inhibited the ANCE-induced release of IL-6, IL-8, and MCP-1 from ARPE-19 cells, suggesting that IL-1 signaling contributes to necrosis-triggered inflammation in the RPE. Neutralization of IL-1α was previously shown to inhibit IL-6 and IL-8 production in human intestinal fibroblasts induced by necrotic cell extracts [30]. Both IL-1α and IL-1β are endogenous mediators of the response to cell injury, with IL-1β being activated by caspase-1 in a manner dependent on inflammasome formation [31]. We have now shown that a pan-caspase inhibitor, Z-VAD-FMK, did not affect ANCE-induced cytokine and chemokines release from ARPE-19 cells, suggesting that IL-1β does not participate in this process. In contrast, pro–IL-1α is processed to mature IL-1α by calpain [23]. We detected the mature form of IL-1α in ANCE. In addition, we found that recombinant human IL-1α stimulated IL-6, IL-8, and MCP-1 release by ARPE-19 cells. Our results thus suggest that IL-1α functions as a danger signal released from necrotic RPE cells and plays a key role in the sterile inflammatory response in the RPE. IL-1α activity is controlled in a cell type–specific manner [23]. The mechanism by which pro–IL-1α is cleaved and activated in necrotic RPE cells requires further investigation.

NF-κB is a key transcriptional activator of many proinflammatory genes. In its inactive state, NF-κB is restricted to the cytosol through its association with the inhibitory protein IκB. Cell stimulation with various signals induces the phosphorylation of IκB by IκB kinase (IKK) and its consequent ubiquitylation and degradation by the proteasome [32]. The released NF-κB then translocates to the nucleus, binds to its target gene promoters, and activates gene transcription. In the present study, we found that ANCE induced activation of NF-κB signaling in ARPE-19 cells and that the ANCE-induced release of IL-6, IL-8, and MCP-1 from these cells was blocked by an IKK2 inhibitor. Necrotic macrophages or liver homogenate induced rapid phosphorylation and degradation of IκB in macrophages, whereas liver homogenate lacking IL-1α induced a markedly reduced level of NF-κB activation in these cells [13]. Our results thus implicate NF-κB in the ANCE-induced up-regulation of proinflammatory cytokine and chemokine expression in RPE cells.

We also found that HNCE induced the release of IL-6 and IL-8, but not that of MCP-1, from HCE cells, suggesting that the pattern of cytokine and chemokine expression induced by necrotic cell extracts may differ among cell types. IL-1α has been shown to be a key danger signal released from necrotic cells to trigger inflammation in several other cell types [13,23]. Multiple signaling pathways including those mediated by protein kinase C, tyrosine phosphorylarion, and an independent third mechanism have been found to regulate activation of the MCP-1 gene [33]. The activation of different signaling pathways or a difference in components of necrotic cell extracts may thus contribute to the difference in necrosis-induced MCP-1 expression between ARPE-19 and HCE cells.

A limitation of our study is that it is based on the ARPE-19 cell line rather than on primary RPE cells. ARPE-19 is a spontaneously transformed line of human RPE cells that appears to possess a normal karyotype. However, properties of ARPE-19 cells, such as the function of tight junctions, appear to be dependent on culture conditions [34], and differences in gene expression between ARPE-19 cells and native RPE cells have been described [35]. Further examination of the effects of necrosis on native RPE cells and on retinal inflammation in vivo is warranted.

In conclusion, our results suggest that IL-1α is an important danger signal that is released from necrotic RPE cells and triggers the secretion of proinflammatory cytokines and chemokines from intact RPE cells in a manner dependent on NF-κB signaling. IL-1α is therefore a potential therapeutic target for amelioration of retinal inflammation.
